# Firearm injuries in Missouri

**DOI:** 10.1371/journal.pone.0294737

**Published:** 2023-11-22

**Authors:** Frederick P. Rivara, Ashley B. Hink, Deborah Kuhls, Samantha Banks, Lauren L. Agoubi, Shelbie Kirkendoll, Alex Winchester, Christopher Hoeft, Bhavin Patel, Avery Nathens

**Affiliations:** 1 Departments of Pediatrics and Epidemiology and the Firearm Injury and Policy Research Program, University of Washington, Seattle, Washington, United States of America; 2 Department of Surgery, Medical University of South Carolina, Charleston, South Carolina, United States of America; 3 Department of Surgery, Kirk Kerkorian School of Medicine at University of Nevada Las Vegas, Las Vegas, Nevada, United States of America; 4 Firearm Injury and Policy Research Program, University of Washington, Seattle, Washington, United States of America; 5 Harborview Injury Prevention and Research Center and the Department of Surgery, University of Washington, Seattle, Washington, United States of America; 6 Department of Surgery, Northwestern Feinberg School of Medicine, Chicago, Illinois, United States of America; 7 American College of Surgeons, Chicago, Illinois, United States of America; 8 Sunnybrook Health Sciences Center, University of Toronto, Toronto, Ontario, Canada; Emory University School of Medicine, UNITED STATES

## Abstract

Firearm deaths continue to be a major public health problem, but the number of non-fatal firearm injuries and the characteristics of patients and injuries is not well known. The American College of Surgeons Committee on Trauma, with support from the National Collaborative on Gun Violence Research, leveraged an existing data system to capture lethal and non-lethal injuries, including patients treated and discharged from the emergency department and collect additional data on firearm injuries that present to trauma centers. In 2020, Missouri had the 4th highest firearm mortality rate in the country at 23.75/100,000 population compared to 13.58/100,000 for the US overall. We examined the characteristics of patients from Missouri with firearm injuries in this cross-sectional study. Of the overall 17,395 patients, 1,336 (7.7%) were treated at one of the 11 participating trauma centers in Missouri during the 12-month study period. Patients were mostly male and much more likely to be Black and uninsured than residents in the state as a whole. Nearly three-fourths of the injuries were due to assaults, and overall 7.7% died. Few patients received post-discharge services.

## Introduction

Firearm injuries and deaths continue to be a major public health problem, resulting in 48,830 deaths in the US in 2021, of which 53.8% were suicides and 42.9% were homicides [1]. However, the number of non-fatal firearm injuries and the characteristics of patients and injuries is not well known, as documented by a recent report from NORC at the University of Chicago [2]. The existing databases do not collect or report on clinical information such as severity of injuries and their outcomes, nor do they provide data that better contextualize injuries including community characteristics, individual risk factors, co-morbid illnesses, substance abuse or mental illness, life stressors, prior violent injuries or suicide attempts, how or why firearms are accessed or obtained for suicide attempts, circumstances preceding injury, and victim-perpetrator relationships. It is recognized that these data are critically important to provide insights into *why* such injuries occur, what are modifiable risk factors, and potential interventions. For this reason, mechanisms to capture these data have been developed through the CDC National Violent Death Reporting System (NVDRS) but are limited to injuries that result in death [3]. Given the large proportion of firearm injuries that are non-lethal (approximately 89% of firearm assaults and unintentional shootings, although only about 10% of suicide attempts) [4], this is a significant limitation and has the potential to significantly bias our understanding of risk factors and the circumstances surrounding the firearm injury event, therefore limiting our understanding of potential interventions to prevent re-injury or death.

To address these needs, the American College of Surgeons Committee on Trauma (ACS COT), with support from the National Collaborative on Gun Violence Research (NCGVR), leveraged an existing data system to capture non-lethal injuries, including patients treated and discharged from the emergency department and collect additional data on firearm injuries that present to trauma centers in the U.S.

The NCGVR asked the research team to perform sub-group analysis of the data collected on firearm injuries treated in trauma centers in the state of Missouri. As of 2020, the rate of firearm deaths in Missouri had risen 70% over the prior 10 years compared to 33% across the country [1]. Additionally, In 2020, Missouri had the 4th highest firearm mortality rate in the country at 23.75/100,000 population compared to 13.58/100,000 for the US as a whole, and, in the same year, St. Louis had the highest murder rate of any large city in the US at 66/100,000.

## Methods

The ACS Trauma Quality Improvement Program (TQIP) collects data for the purposes of performance benchmarking from over 700 centers, representing more than 800 distinct trauma programs, across the U.S., capturing an estimated 90%-95% of all level 1 and 2 (major trauma centers) and a less comprehensive sample of level 3 trauma centers. Prior studies indicate that verified and/or designated trauma centers care for approximately 70% of medically treated firearm injuries in the U.S [5]. Data collected are specified in the National Trauma Data Standard [6] and include patient and injury characteristics, processes of care and outcomes among all patients admitted, transferred to that center, or who died in hospital. Notably, prior to this initiative, the ACS TQIP only collected data on people with firearm injuries who met these criteria; patients assessed in the emergency department and discharged home were not routinely captured.

We recruited volunteer participation from the entire TQIP sample and provisionally enrolled 165 trauma centers to participate in this program; 128 ultimately contributed data, of which 11 were in Missouri. Centers had to participate in ACS TQIP over the duration of the study and agreed to collect data elements listed below on all individuals treated with firearm injuries (including those discharged alive from the emergency department). Centers were not provided any funding to support additional data collection. Centers were recruited through electronic mailings to the trauma directors in all ACS TQIP centers, holding a webinar for potential centers, ACS COT newsletters, and direct contact by study investigators with trauma directors.

### Study population

Patients eligible for the study were individuals of any age arriving alive at a participating trauma center in Missouri and with residential zip codes in Missouri between March 1, 2021 and February 28, 2022 who had sustained an injury due to a firearm. The study was approved by Advarr CIRBI^TM^. Consent for the registry to abstract data from the medical record was waived by the IRB. Centers started participation at varying times during the study period and may not have contributed 12-months of data.

### Data abstraction

Data were abstracted from the electronic health record by trained personnel at each trauma center including registrars, clinicians, and research staff. In addition to the standard data collected for TQIP, the abstractors also extracted available additional data specific to this study. This new data included (See S1 File):

- Demographic characteristics: education, veteran status, and caregivers (pediatric patients)
- Risk factors: illicit substance use and intoxication, history of or newly identified/diagnosed mental illness, prior arrests/involvement in the criminal justice system, Adverse Childhood Experiences.
- Circumstances: Context/preceding events (*assaults*: altercation, commission of a crime, drug or gang-related, bystander, mass violence, intimate partner violence, child abuse; *suicide attempts*: life stressors, declining mental illness, terminal medical illness, suicide-homicides; *unintentional*: playing, cleaning, handling, hunting, accidental discharge when unaware of firearm presence), specific location of injury occurrence, perpetrator-victim relationships.
- Firearms (for self-inflicted and unintentional injuries): type implicated in the injury, ownership, access and storage at time of the incident (for self-inflicted and unintentional injuries).
- Functional outcomes, medical needs and services at time of discharge.

The direction provided to participating centers was to abstract these data from the EMS, emergency department, or inpatient medical record. There was no expectation for additional interview of patients to capture data that was not otherwise routinely collected during the course of care. Together, this study required two modifications to existing data abstraction for trauma centers: 1) expanded inclusion criteria to capture patients discharged from the ED to include those that may not have qualified for TQIP inclusion and 2) expanded data abstraction to include additional information on the context of firearm injuries.

### Data analysis

Sex was imputed since the original variable had a high proportion of missingness among patients due to abstraction error at two centers. The alcohol screen result was also imputed for all patients (originally 17.7% missing overall).

Race, mental illness, injury intent, drug screen, and discharge services were all variables in which more than one choice could be selected by the coders and were recoded to ease interpretation. Patients who were reported to have more than one racial category were grouped together. Injury intent was originally a variable in which a patient could have multiple intents, and the context of injury variable was used to help inform the mutually exclusive categories used in these tables.

Drug screen results were also reported here in mutually exclusive categories. Patients positive for only one drug were represented in the relevant category. A separate category for patients who were positive for cannabinoid in addition to any other drug listed was created, in addition to a separate category for patients who were positive for more than one drug excluding cannabinoid.

Post-discharge service variables (rehabilitation/post-discharge needs, home health needs, and psychosocial ancillary services) were also “select all that apply” variables; within each post-discharge service variable, those with more than one service were counted in each category that was applicable (non-mutually exclusive categories). A variable was also created to indicate whether a patient had any post-discharge service in any of the three service categories (rehabilitation/post-discharge needs, home health needs, and psychosocial ancillary services) vs. none.

An urbanicity variable was created to further explore the comparisons between Missouri patients and patients in other states. By linking the Rural-Urban Commuting Codes (RUCA) [7] via ZIP code to our patient data, we categorized zip codes of their residence with a RUCA code of 1 as urban, and all other codes (2–10) as rural.

To provide additional community context around the patient and injury, we linked data from the Distressed Community Index (DCI) to patient records meeting our inclusion criteria via ZIP code. The DCI is a validated index of prosperity that includes variables related to education, housing, unemployment, poverty, and changes in business establishments [8]. The scale ranges from 0–100 and is sorted into quintiles with the highest scores representing the most distressed communities. DCI is not calculated for ZIP codes with less than 500 residents. DCI data is publicly available and utilizes the U.S. Census Bureau’s American Community Survey 5- Year Estimates as well as the Census Bureau’s Business Pattern’s dataset for 2016 and 2020.

P-values were calculated using Pearson’s Chi Squared tests for mutually exclusive categorical variables and two-sided t-tests for numerical variables. In cases where there were fewer than 10 observations per cell, Fisher’s exact tests were used. P-values were considered significant at the 0.05 alpha level. All data cleaning and tables were done in RStudio 4.2.2.

## Findings

### Demographic factors

Data were collected on 17,395 patients of whom 1,336 (7.7%) were treated at one of the 11 participating trauma centers in Missouri. As shown in Table 1, these patients were mostly young (median age 29.0) adult males (83.0%). Firearm injured patients in Missouri were much more likely to be Black (78.8% compared with 11.8% of state residents) and much less likely to be White (15.0% compared with 82.5% of state residents). More than half of the patients in Missouri were recorded as being uninsured, compared to 9.3% in the state as a whole. The majority (72.9%) of firearm injuries were due to assaults with few due to self-inflicted injuries, since most such firearm injuries result in death at the scene.

**Table 1:** Socio-demographic characteristics of firearm injury patients with Missouri zip codes.

	Firearm patients (N = 1336)	Missouri residents
**Age (years)**		
Median	29.0	39.1
Missing	1 (0.1%)	“
**Sex (Missing Values Imputed)**		
Female	227 (17.0%)	50.6%
Male	1109 (83.0%)	49.4%
Non-Binary	0 (0%)	
**Race**		
American Indian	1 (0.1%)	0.6%
Asian	3 (0.2%)	2.3%
Black	1053 (78.8%)	11.7%
Other	9 (0.7%)	11
Pacific Islander	2 (0.1%)	0.2%
White	201 (15.0%)	82.5%
>1 Race	2 (0.1%)	2.7%
Missing	65 (4.9%)	—
**Ethnicity**		
Hispanic/Latino	17 (1.3%)	4.8%
Not Hispanic/Latino	1236 (92.5%)	95.2%
Missing	83 (6.2%)	—
**Primary Payment Method**		
Medicaid/Other Government	363 (27.2%)	14.7%
Medicare	54 (4.0%)	16.4%
Private/Commercial Insurance	199 (14.9%)	59.6%
Uninsured	709 (53.1%)	9.3%
Missing	11 (0.8%)	—
**Injury Intent**		
Assault	974 (72.9%)	
Law Enforcement	11 (0.8%)	
Self-Inflicted	52 (3.9%)	
Unintentional	144 (10.8%)	
Missing	155 (11.6%)	
**Context of Injury** ^**1**^		
** Assault**	**542 (40.6%)**	
Community Violence	255 (47.0%)	
Bystander	63 (11.6%)	
Interpersonal	105 (19.4%)	
Drug Related	8 (1.5%)	
Intimate Partner	14 (2.6%)	
Family Violence	12 (2.2%)	
Mass Shooting	9 (1.7%)	
Random^2^	75 (13.8%)	
Hate Crime	0 (0%)	
Intervening	2 (0.4%)	
Line of Duty	2 (0.4%)	
Commission of Crime	12 (2.2%)	
Sexual Assault	1 (0.2%)	
Robbery	33 (6.1%)	
**Self**	**31 (2.3%)**	
Intoxication	8 (25.8%)	
Mental Illness	15 (48.4%)	
Cognitive Impairment	1 (3.2%)	
Medical Condition	4 (12.9%)	
Personal Crisis	14 (45.2%)	
Murder Suicide	3 (9.7%)	
** Unintentional**	**135 (10.1%)**	
Handling	106 (78.5%)	
Playing	11 (8.1%)	
Hunting	3 (2.2%)	
Accidental	11 (8.1%)	
Celebration	0 (0%)	
Sport	4 (3.0%)	
Training	0 (0%)	
** Missing**	**628 (47.0%)**	

1. Subcategories are not mutually exclusive, and percentages calculated among patients where parent variable was reported as present.

2. Random “indicates an act in which the suspect is not concerned with who is being harmed, just that someone is being harmed, such as a person who shoots randomly at passing cars from a highway bridge.”

In the 708 patients for whom context of injury data was available, assault injuries most commonly occurred in the context of community violence. Shootings related to interpersonal and random violence were also common. Self-inflicted injuries occurred in the context of intoxication in 25.8% patients, and were associated with a personal crisis in almost half, while mental illness was reported in 48.4%. Unintentional injuries were usually associated with handling the firearm.

### Risk factors for firearm injury

We examined a number of factors, as recorded in the medical record, which are known to be associated with firearm injuries as shown in Table 2. About 1 in 6 patients was intoxicated at the time of the emergency department (ED) assessment. Drug testing was not done on 71.8% of patients in Missouri, but among those tested only 22% were negative for any drugs. The most common drug found on toxicology assessment was cannabis. Prior mental illness was recorded in 12.6% of patients but was missing in 29.0%. The injuries occurred in a variety of settings, most commonly in homes, streets, motor vehicles and commercial areas. Nearly three-fourths (72.2%) of patients with prior assault injuries had sustained a prior gunshot wound.

**Table 2:** Pre-injury factors of firearm injury patients with Missouri zip codes.

	N (%) (N = 1336)
**Alcohol Screen Result**	
< = 0.08 BAC	1119 (83.8%)
> 0.08 BAC	217 (16.2%)
**Drug Screen**	
Negative for any drugs	83 (6.2%)
Amphetamine	15 (1.1%)
Benzodiazepines	5 (0.4%)
Cannabinoid	153 (11.5%)
Cocaine	16 (1.2%)
Methamphetamine	0 (0%)
Opioids	4 (0.1%)
Phencyclidine	0 (0%)
Tricyclic Antidepressants	0 (0%)
Other	0 (0%)
Cannabinoid and 1 other drug	82 (6.1%)
>1 drug (other than cannabinoid)	19 (1.4%)
Missing	959 (71.8%)
**Mental Illness**	
None	779 (58.3%)
Any Mental Illness	169 (12.6%)
Missing	388 (29.0%)
**Injury Setting**	
Residence	376 (28.1%)
Street	390 (29.2%)
Motor Vehicle (other than public transport)	180 (13.5%)
Public Transit	7 (0.5%)
Commercial Area	137 (10.3%)
School	0 (0%)
Natural Area	31 (2.3%)
Other	1 (0.1%)
Missing	214 (16.0%)
**Previous Violent Assaults/Injuries**	
None	447 (33.5%)
Any	158 (11.8%)
Gunshot Wound	114 (72.2%)
Knife Stabbing	9 (5.7%)
Sexual Assault	3 (1.9%)
Blunt object Injury/Assault	39 (24.7%)
Strangulation or Suffocation	1 (0.6%)
Missing	731 (54.7%)
**Previously/Currently under arrest/incarcerated**	
Yes	51 (3.8%)
No	407 (30.5%)
Missing	878 (65.7%)

Subcategories are not mutually exclusive, and percentages calculated among patients where parent variable was reported

### Characteristics of treatment

Overall, 43.4% of firearm patients treated at these trauma centers were discharged from the hospital alive; 7.7% of patients presenting to trauma centers in Missouri with firearm injuries died in the hospital, with 66 of the 103 fatalities dying in the ED (Table 3). Only 12.3% of patients received services on discharge from the hospital; only 4.3% received social work or mental health services and only 5.4% received any services related to violence.

**Table 3:** Hospital course and outcomes of Missouri firearm injury patients.

	N (%) (N = 1336)
**Mortality**	
Deceased/Transferred to Hospice	103 (7.7%)
Survived	1233 (92.3%)
**Injury Severity Score**	
Mean (SD)	8.91 (10.6)
Median [Min, Max]	5.00 [1.00, 75.0]
Missing	27 (2.0%)
**ED Discharge Disposition**	
Discharged Alive/Left against advice	580 (43.4%)
Admitted to the OR	333 (24.9%)
Admitted to the ICU	110 (8.2%)
Admitted to floor/observation unit/Telemetry/Step-down unit	208 (15.6%)
Died in the ED	66 (4.9%)
Transferred to another facility	9 (0.7%)
Other^1^	7 (0.5%)
Missing	23 (1.7%)
**Hospital Discharge Disposition (among admitted)**	
Home	506 (75.5%)
SNF or other long-term care	77 (11.4%)
Transferred to acute care	4 (0.6%)
Other^2^	46 (6.8%)
Died	37 (5.5%)
Missing	4 (0.6%)
**Number of Discharge Services among those discharged alive**	N = 1233
None	1066 (86.5%)
Missing^3^	15 (1.2%)
Any Discharge Care	152 (12.3%)
**Rehabilitation/Post-Discharge Needs** ^**4**^	
Inpatient Rehab	80 (6.5%)
Outpatient Physical/Occupational Therapy	20 (1.6%)
Outpatient Speech Therapy	2 (0.2%)
Outpatient Rehab	1 (0.1%)
**Home Health Needs** ^**4**^	
Nursing	28 (2.3%)
Wound Care	27 (2.2%)
Other	14 (1.1%)
**Psychosocial Ancillary Services** ^**4**^	
Social Work/Mental Health Services	53 (4.3%)
Violence Intervention and IPV Services	67 (5.4%)
Other	23 (1.9%)

1. Jail, institutional care, mental health, etc.

2. Discharged/transferred to court/law enforcement, left against medical advice or discontinued care

3. Patients who are missing information about all post-discharge needs.

4. Percentages calculated among surviving patients (N = 1233). Patients receiving more than one post-discharge service are represented in all relevant categories (not mutually-exclusive categories).

We also examined how these treatments varied across age groups (Table 4). Few of those 65 and older were discharged from the ED, and more this age required skilled nursing facility (SNF) or other long-term care on discharge from the hospital. Those in the middle age group were significantly less likely to receive any services on discharge from the hospital.

**Table 4:** Hospital course and outcomes of firearm injury patients of firearm patients by age group.

	Pediatric (<20)	Adult (20–65)	Older Adult (>65)	P-value
	N = 226	N = 1081	N = 28	
**Mortality**				
Deceased/Transferred to Hospice	12 (5.3%)	89 (8.2%)	1 (3.6%)	0.281
Survived	214 (94.7%)	992 (91.8%)	27 (96.4%)	
**Injury Severity Score**				
Mean (SD)	7.75 (8.92)	9.09 (10.9)	11.1 (9.54)	0.132
Median	4.00	5.00	9.00	
[Min, Max]	[1.00, 43.0]	[1.00, 75.0]	[1.00, 32.0]	
Missing	12 (5.3%)	15 (1.4%)	0 (0%)	
**ED Discharge Disposition**				
Discharged Alive/Left against advice	98 (43.4%)	479 (44.3%)	3 (10.7%)	<0.001
Admitted to the OR	52 (23.0%)	275 (25.4%)	6 (21.4%)	
Admitted to the ICU	20 (8.8%)	87 (8.0%)	3 (10.7%)	
Admitted to floor/observation unit/Telemetry/Step-down unit	42 (18.6%)	155 (14.3%)	11 (39.3%)	
Died in the ED	7 (3.1%)	58 (5.4%)	0 (0%)	
Transferred to another facility	4 (1.8%)	5 (0.5%)	0 (0%)	
Other^1^	0 (0%)	5 (0.5%)	2 (7.1%)	
Missing	3 (1.3%)	17 (1.6%)	3 (10.7%)	
**Hospital Discharge Disposition (among admitted)**				
Home	94 (80.3%)	401 (75.1%)	11 (47.8%)	<0.001
SNF or other long-term care	9 (7.7%)	60 (11.2%)	8 (34.8%)	
Transferred to acute care	1 (0.9%)	1 (0.2%)	2 (8.7%)	
Other^2^	5 (4.3%)	40 (7.5%)	1 (4.3%)	
Died	5 (4.3%)	31 (5.8%)	1 (4.3%)	
Missing	3 (2.6%)	1 (0.2%)	0 (0%)	
**Number of Discharge Services for those discharged alive**				
None	146 (64.6%)	903 (83.5%)	17 (60.7%)	<0.001
Any Discharge Care	65 (30.4%)	78 (7.9%)	9 (33.3%)	
Missing	3 (1.3%)	11 (1.0%)	1 (3.6%)	

1. Jail, institutional care, mental health, etc.

2. Discharged/transferred to court/law enforcement, left against medical advice or discontinued care

When we examined patient outcomes by insurance status, stratifying for injury intent, there were no significant differences in mortality (Table 5). However, those who were injured by assault and covered by Medicaid or Medicare were less likely to be discharged home from the ED compared to those in the other two insurance groups. Those with commercial insurance had the lowest likelihood of receiving any post-discharge services.

**Table 5:** Patient outcomes by insurance status and injury intent.

	Medicaid/Medicare/Other Government			Commercial			Uninsured		
	Assault (N = 312)	Unintentional (N = 47)	p-value	Assault (N = 135)	Unintentional (N = 36)	p-value	Assault (N = 532)	Unintentional (N = 61)	p-value
**Age (years)**									
Mean (SD)	28.8 (13.8)	31.6 (22.9)	<0.001	29.2 (11.4)	32.2 (12.8)	0.204	32.2 (9.97)	31.6 (10.7)	0.269
Median [Min, Max]	25.0 [3.00, 86.0]	21.0 [2.00, 74.0]		26.0 [14.0, 70.0]	28.0 [14.0, 60.0]		30.0 [11.0, 73.0]	30.0 [6.00, 58.0]	
Missing	0 (0%)	0 (0%)		0 (0%)	0 (0%)		1 (0.2%)	0 (0%)	
**Mortality**									
Deceased/Transferred to Hospice	14 (4.5%)	0 (0%)	0.003	5 (3.7%)	0 (0%)	0.004	53 (10.0%)	0 (0%)	<0.001
Survived	298 (95.5%)	47 (100%)		130 (96.3%)	36 (100%)		479 (90.0%)	61 (100%)	
**ED Discharge Disposition**									
Discharged Alive/Left against advice	110 (35.3%)	16 (34.0%)	<0.001	54 (40.0%)	22 (61.1%)	<0.001	263 (49.4%)	39 (63.9%)	<0.001
Admitted to the OR	91 (29.2%)	14 (29.8%)		39 (28.9%)	6 (16.7%)		122 (22.9%)	9 (14.8%)	
Admitted to the ICU	35 (11.2%)	2 (4.3%)		15 (11.1%)	2 (5.6%)		29 (5.5%)	1 (1.6%)	
Admitted to floor/observation unit/Telemetry/Step-down unit	60 (19.2%)	13 (27.7%)		23 (17.0%)	5 (13.9%)		69 (13.0%)	9 (14.8%)	
Died in the ED	8 (2.6%)	0 (0%)		3 (2.2%)	0 (0%)		38 (7.1%)	0 (0%)	
Transferred to another facility	4 (1.3%)	0 (0%)		0 (0%)	1 (2.8%)		2 (0.4%)	0 (0%)	
Other ^1^	2 (0.6%)	0 (0%)		0 (0%)	0 (0%)		2 (0.4%)	0 (0%)	
Missing	2 (0.6%)	2 (4.3%)		1 (0.7%)	0 (0%)		7 (1.3%)	3 (4.9%)	
**Hospital Discharge Disposition (among admitted)**	N = 188	N = 31		N = 78	N = 13		N = 227	N = 22	
Home	138 (73.4%)	29 (93.5%)	<0.001	64 (82.1%)	10 (76.9%)	0.028	177 (78.0%)	21 (95.5%)	<0.001
SNF or other long-term care	29 (15.4%)	1 (3.2%)		9 (11.5%)	1 (7.7%)		12 (5.3%)	0 (0%)	
Transferred to acute care	0 (0%)	0 (0%)		0 (0%)	0 (0%)		1 (0.4%)	0 (0%)	
Other ^2^	14 (7.4%)	0 (0%)		3 (3.8%)	2 (15.4%)		21 (9.3%)	1 (4.5%)	
Died	6 (3.2%)	0 (0%)		2 (2.6%)	0 (0%)		15 (6.6%)	0 (0%)	
Missing	1 (0.5%)	1 (3.2%)		0 (0%)	0 (0%)		1 (0.4%)	0 (0%)	
**Number of Discharge Services (among surviving)**	N = 289	N = 47		N = 130	N = 36		N = 479	N = 61	
No Discharge Care	228 (76.5%)	36 (76.6%)	0.002	118 (90.8%)	34 (94.4%)	<0.001	446 (93.1%)	54 (88.5%)	<0.001
Any Discharge Care	69 (23.2%)	9 (19.1%)		12 (9.2%)	1 (2.8%)		26 (5.4%)	6 (9.8%)	
Missing	1 (0.3%)	2 (4.3%)		0 (0%)	1 (2.8%)		7 (1.5%)	1 (1.6%)	

1. Jail, institutional care, mental health, etc.

2. Discharged/transferred to court/law enforcement, left against medical advice or discontinued care

Patients with a “Law Enforcement” injury intent were combined with “Assault.”

### Socioeconomic community factors

Patients with firearm injuries in Missouri lived in communities with much higher DCI scores and were much more likely to live in the most distressed communities (69.4% vs 24.6%) compared to the state as a whole (Table 6). Older adults were less likely to live in the highest DCI quintile compared to those 65 and younger (42.9% vs 70.0%). Patients who sustained firearm injuries from assault had the highest mean DCI and were most likely to live in the most distressed communities. Patients in urban areas were nearly twice as likely to come from the most distressed quintile compared to those living in rural areas.

**Table 6:** DCI by patient characteristics.

	Mean (SD)	DCI Quintiles N (%)^1^				
		Q1	Q2	Q3	Q4	Q5
State of Missouri	61.5 (29.1)	24.0%	15.1%	14.9%	20.3%	24.6%
All	81.1 (24.8)	61 (4.6%)	73 (5.5%)	104 (7.8%)	161 (12.1%)	927 (69.4%)
Age group						
<20	82.7 (22.9)	7 (3.1%)	13 (5.8%)	17 (7.5%)	27 (11.9%)	161 (71.2%)
20–65	81.2 (24.9)	49 (4.5%)	58 (5.4%)	83 (7.7%)	128 (11.8%)	754 (69.8%)
>65	65.0 (32.9)	5 (17.9%)	2 (7.1%)	3 (10.7%)	6 (21.4%)	12 (42.9%)
Injury intent						
Assault	82.2 (24.0)	37 (3.8%)	54 (5.5%)	70 (7.2%)	114 (11.7%)	695 (71.4%)
Law enforcement	67.5 (29.7)	1 (9.1%)	1 (9.1%)	2 (18.2%)	2 (18.2%)	5 (45.5%)
Self-inflicted	63.6 (29.4)	8 (15.4%)	4 (7.7%)	5 (9.6%)	15 (28.8)%	17 (32.7)%
Unintentional	73.3 (29.3)	13 (9.0%)	8 (5.6%)	18 (12.5%)	19 (13.2%)	83 (57.6%)
Rural	70.7 (25.6)	6 (5.8%)	8 (7.8%)	15 (14.6%)	26 (25.2%)	41 (39.8%)
Urban	81.9 (24.6)	55 (4.5%)	65 (5.3%)	89 (7.2%)	135 (10.9%)	886 (71.9%)

### Rural and urban differences

Using the RUCA codes for residence zip code, 7.7% of the patients were from rural Missouri. Firearm injured patients living in rural areas were somewhat older than those in urban areas (Table 7). They were much less likely to be Black compared to patients in urban areas (18.4% vs 83.9%) Patients living in urban areas were more likely to be uninsured. Three-quarters of patients in urban areas were injured in assaults compared with less than half in rural areas. Fewer patients in urban areas received any post-discharge services.

**Table 7:** Sociodemographic, pre-injury, and community characteristics of firearm injury patients by urbanicity.

	Rural (N = 103)	Urban (N = 1233)	p-value
**Age (years)**			
Mean (SD)	35.5 (16.1)	30.7 (12.4)	0.004
Median [Min, Max]	33.0 [2.00, 76.0]	29.0 [0, 86.0]	
Missing	0 (0%)	1 (0.1%)	
**Sex**			
Female	16 (15.5%)	211 (17.1%)	0.785
Male	87 (84.5%)	1022 (82.9%)	
Non-Binary	0 (0%)	0 (0%)	
**Race**			
American Indian	0 (0%)	1 (0.1%)	<0.001
Asian	0 (0%)	3 (0.2%)	
Black	19 (18.4%)	1034 (83.9%)	
Other	3 (2.9%)	6 (0.5%)	
Pacific Islander	1 (1.0%)	1 (0.1%)	
White	72 (69.9%)	129 (10.5%)	
>1 Race	1 (1.0%)	1 (0.1%)	
Missing	7 (6.8%)	58 (4.7%)	
**Ethnicity**			
Hispanic/Latino	3 (2.9%)	14 (1.1%)	0.129
Not Hispanic/Latino	91 (88.3%)	1145 (92.9%)	
Missing	9 (8.7%)	74 (6.0%)	
**Primary Payment Method**			
Medicaid/Other Government	30 (29.1%)	333 (27.0%)	0.005
Medicare	10 (9.7%)	44 (3.6%)	
Private/Commercial Insurance	19 (18.4%)	180 (14.6%)	
Self-Pay/Not Billed/Other	43 (41.7%)	666 (54.0%)	
Missing	1 (1.0%)	10 (0.8%)	
**Injury Intent**			
Assault	49 (47.6%)	925 (75.0%)	<0.001
Law Enforcement	2 (1.9%)	9 (0.7%)	
Self-Inflicted	23 (22.3%)	29 (2.4%)	
Unintentional	28 (27.2%)	116 (9.4%)	
Missing	1 (1.0%)	154 (12.5%)	
**Injury Severity Score**			
Mean (SD)	11.3 (11.2)	8.70 (10.5)	0.024
Median [Min, Max]	9.00 [1.00, 75.0]	4.00 [1.00, 75.0]	
Missing	1 (1.0%)	26 (2.1%)	
**Mortality**			
Deceased/Transferred to Hospice	7 (6.8%)	96 (7.8%)	0.865
Survived	96 (93.2%)	1137 (92.2%)	
**ED Discharge Disposition**			
Discharged Alive/Left against advice	21 (20.4%)	559 (45.3%)	<0.001
Admitted to the OR	26 (25.2%)	307 (24.9%)	
Admitted to the ICU	19 (18.4%)	91 (7.4%)	
Admitted to floor/observation unit/Telemetry/Step-down unit	25 (24.3%)	183 (14.8%)	
Died in the ED	2 (1.9%)	64 (5.2%)	
Transferred to another facility	0 (0%)	9 (0.7%)	
Other^1^	0 (0%)	7 (0.6%)	
Missing	10 (9.7%)	13 (1.1%)	
**Hospital Discharge Disposition (among admitted)**			
Home	55 (68.8%)	451 (75.9%)	0.246
SNF or other long-term care	14 (17.5%)	63 (10.6%)	
Transferred to acute care	1 (1.3%)	3 (0.5%)	
Other^2^	4 (5.0%)	42 (7.1%)	
Died	5 (6.3%)	32 (5.4%)	
Missing	1 (1.3%)	3 (0.5%)	
**Number of Discharge Services** ^**3**^	N = 96	N = 1137	
None	74 (77.1%)	992 (87.2%)	0.0216
Any Discharge Care	19 (19.8%)	133 (11.7%)	
Missing	3 (3.1%)	12 (1.1%)	

1. Jail, institutional care, mental health, etc.

2. Discharged/transferred to court/law enforcement, left against medical advice or discontinued care

3. Percentages calculated among surviving patients. Patients receiving more than one post-discharge service are represented in all relevant categories.

## Discussion

In this analysis of 1336 patients with Missouri residential zip codes treated at 11 trauma centers in Missouri, patients were mostly male and much more likely to be Black and uninsured than residents in the state as a whole. Nearly three-fourths of the injuries were due to assaults, and overall 7.7% died. Few patients received post-discharge services.

A striking finding is the much higher levels of distress in communities in which individuals are experiencing firearm injuries compared to residents of the state as a whole. This is especially true for injuries among patients in urban areas. While firearm injuries have long been associated with poverty, urban areas in Missouri with firearm injuries represent extremely distressed and deprived communities. Combined with the fact that the vast majority of firearm injuries in these urban areas were to Black males, the data demonstrate the effects of long-standing structural racism on the epidemiology of firearm injuries [9–11].

In this study, only 3.8% had a prior arrest although 114 of the total (11.8%) had a prior assault. Given the high degree of socioeconomic disadvantage of the areas in which these patients lived, this is surprising compared to other studies which have found half of firearm injured patients had an arrest in the prior 2 years [12]. This difference may be due to the large amount of missing data in our study for prior assaults and prior arrests.

There was a marked dearth of services for the people being discharged alive from the hospital, especially those with injuries due to assaults. Very few had social work services post-discharge, and even fewer had access to violence prevention programs. Such services are critically important in order to both prevent injury recidivism as well as to help patients and families recover from their trauma [13]. More than one-half of survivors of firearm violence develop Post Traumatic Stress Disorder and/or depression [14] and need interventions starting in trauma centers [15]. It should start with inpatient screening and intervention followed by transition to outpatient care. Without any interventions after discharge, individuals hospitalized with firearm injuries have a 30-fold greater chance of being re-hospitalized with a firearm injury and a 7.3-fold greater chance of dying from a firearm injury than those in the general population [16]. While hospital-based violence intervention programs are often suggested as interventions to prevent recidivism and subsequent violence [17], the currently available evidence suggests otherwise [18, 19].

One of the concerns for firearm injured patients in Missouri is that a large proportion of firearm injured patients are not insured, which can result in further barriers to obtaining follow-up care. While Missouri expanded Medicaid in 2021, the high percentage of firearm injured patients without insurance is of great concern given the potential benefits of Medicaid expansion that include reduction of suicide rates and improving access to inpatient rehabilitation after acute hospitalization among those who are injured by firearms [20, 21].

This study has a number of important implications. The amount of missing data for variables that can be used to guide prevention efforts was large. We do not think this was a failure of chart abstraction by the coders but lack of collection and documentation of this data by health care providers. Efforts to direct resources for primary and second prevention in communities will require that health systems collect this information. Health systems also need to view firearm-injured patients different than other trauma patients, for example those injured in motor vehicle crashes. The high rate of recidivism and risk of subsequent violence and violent death requires that health systems develop, test and implement effective programs to respond to this problem. The clear association with firearm injuries and deaths with socioeconomic distress and poverty is well-known and will require investment in the most distressed neighborhoods in communities.

### Limitations

This study does have important limitations. First, many patients with firearm injuries die at the scene, especially those who used a firearm to attempt suicide, 90% of which result in death [22]. These individuals and characteristics unique to the patients that experience firearm suicide are thus largely missing from these data, reflected in that only 4.3% of the patients in this study had self-inflicted injuries. Second, while the authors believe that the additional data collected on patients admitted to trauma center hospitals is important to understand the risks and circumstances of their injuries, and significant training of trauma center data personnel occurred, the data missingness may represent inadequate resources, training and practices of the entire healthcare team, all of whom gather medical and social histories and document in the EHR. Additionally, the extra time required of trauma personnel to locate and extract the additional data may have limited their ability to do so, notably during the data collection period when trauma centers reported an increase in trauma volume as a result of the COVID-19 pandemic. In addition, the total number of patients included in this study is an under representation of all the patients treated at participating centers, as centers started participation at varying times during the study period and may not have contributed 12-months of data. The amount of time required to extract data was significant and may have been difficult for centers especially since it occurred when hospitals may have been very busy with patients during the pandemic and no additional funding was available to support this data collection at the center level.

In summary, this study describes patients with firearm injuries living in Missouri and treated at ACS trauma centers, enhancing our knowledge of their injuries, clinical care, characteristics, and injury circumstances. The results emphasize the need for interventions at multiple levels for people living in distressed communities to reduce the burden of firearm-related harms, in addition to highlighting potential opportunities to improve access to care and evidence-informed services among those who survive firearm injuries.

## Supporting information

S1 Checklist STROBE statement—checklist of items that should be included in reports of observational studies. (DOCX)

S1 File Data dictionary. (DOCX)
