# Supplementary material for: Firearm injuries in Missouri
Source: PLoS One. 2023 Nov 22;18(11):e0294737. doi: 10.1371/journal.pone.0294737 (PMC10664957; doi:10.1371/journal.pone.0294737)
Supplement: S1 File — (DOCX) [file pone.0294737.s002.docx]

**Appendix A. Data Dictionary**

**Demographics**

| **Variable** | **Definition** | **Options** | **Further Descriptions (mix of qualitative and categorical)** | **Common EMR Locations for Data** |
| --- | --- | --- | --- | --- |
| **Employment status** | The employment and/or student status of the patient at the time of their injury. | 1. Full-time 2. Part-time 3. Homemaker 4. Unemployed 5. Employed and part-time student 6. Student 7. Retired 8. Unknown or unclear |  | H&P  Initial consult notes  Social work  Physical & occupational therapy |
| **Caregivers**  ***Patient age <20** | The primary caregivers for the patient at the time of injury. These should be the individuals that the patient lives with and provides primary overnight and caregiving. For instance, if the patient is living with a grandparent who provides the primary overnight but his or parent has legal custody, the grandparent is the primary caregiver. | 1. Parents 2. Single-parent 3. Foster parent(s) 4. Grandparent(s) 5. Other family member 6. Other friend/adult 7. State custody 8. Unknown or unclear |  | H&P  Pediatrics  Social work |
| **Veteran status** | This indicates if the patient previously or currently serves in the military. | 1. Yes 2. No 3. Unknown or unclear |  | H&P  Social work  Psychiatry  Psychology |

**Risk Factors**

| **Variable** | **Definition** | **Options** | **Further Descriptions (Qualitative)** | **Common EMR Locations for Data** |
| --- | --- | --- | --- | --- |
| **Illicit substance use** | This indicates if the patient has current illicit substance use. This may include if the patient tests positive at the time of injury or if there is a documented history of illicit substance use within the past 3 months. | 1. Yes (select all that apply)   1. Methamphetamine 2. Marijuana 3. Prescription opiate 4. Heroin 5. Hallucinogenic 6. Benzodiazepine 7. Other   2. No substance use  3. Suspected but unclear  4. Unknown or Not assessed  *Suspected but unclear* indicates that there is clinical suspicion that the patient uses an illicit substance, but is not officially diagnosed or indicated by the patient. |  | Admission urine tox screen  EMS documentation  H&P  Social work  Psychiatry  Psychology  Substance abuse counseling |
| **ETOH abuse and /or dependence** | This indicates if the patient has pre-existing ETOH abuse and/or dependence. | 1. Yes   1. Dependence 2. Abuse   2. No  3. Suspected but unclear  4. Unknown or Not assessed  *Suspected but unclear* indicates that there is clinical suspicion that the patient has a history of ETOH abuse and/or dependence, but is not officially diagnosed or indicated by the patient. |  | Admission blood ETOH screen  EMS documentation  H&P  Social work  Psychiatry  Psychology  Substance abuse counseling |
| **ETOH intoxication** | This indicates if the patient is acutely intoxicated with ETOH at the time of injury (ETOH >0.08%). | 1. Yes  2. No  3. Suspected but BAC not done  4. Unknown  *Suspected but BAC not done* indicates that the patient demonstrated clinical signs of ETOH intoxication in the setting of corroborating history (patient or witnesses reported ETOH consumption), but there was no ETOH level drawn, or the patient was transferred and there were many hours between the time of injury and testing when there is clinical suspicion or history provided that suggests ETOH intoxication. |  | Admission blood ETOH screen  H&P |
| **Psychiatric illness** | This indicates if the patient has a known pre-existing history of mental illness at the time of injury or is diagnosed with a mental illness during hospitalization, or had a prior hospitalization where this was noted. | 1. Yes (select all that apply)   1. Depression 2. Anxiety 3. Bipolar disorder 4. PTSD 5. Schizophrenia 6. Personality disorder 7. Other   2. No  3. Suspected but unclear  4. Unknown or Not assessed  *Suspected but unclear* indicates that it is unclear if the patient carried a formal diagnosis, but the patient endorsed a mental illness or cognitive impairment, or there is strong clinical evidence or concern documented by the clinical team. |  | H&P  Initial consult notes  Psychiatry  Psychology  Social work  *Check problem lists and actual note documentation. Many may not be populated in EMR problem lists or comorbid illnesses in NTDB. |
| **Cognitive impairment** | This indicates if the patient has a pre-existing cognitive impairment at the time of injury or is diagnosed with a cognitive impairment during hospitalization. | 1. Yes  2. No  3. Suspected but unclear  4. Unknown or Not assessed  **Cognitive impairment* indicates if the patient has a known or suspected disorder that impairs cognitive function such as memory, decision-making and judgment not due to acute substance abuse. These could include dementia, neurologic or medical disorders, prior TBI with residual effects, developmental disabilities, and syndromes that impact cognitive development (for example, Down syndrome).  *Suspected but unclear* indicates that it is unclear if the patient carried a formal diagnosis of cognitive impairment, but there is clinical evidence or concern documented by the medical team. |  | H&P  Psychiatry  Internal medicine or geriatric medicine  Social work |
| **Prior arrests and/or incarceration** | This indicates if the patient has been previously incarcerated or arrested, or if the patient is currently under arrest for a crime (not including the incident resulting in the GSW). | 1. Yes  2. No  3. Unknown or Not assessed |  | H&P  Social work  Psychiatry  Psychology |
| **Prior violent injuries** | This indicates if the patient has a history of experiencing a violent injury prior to the one they were hospitalized for. | 1. Yes (select all that apply)   1. GSW 2. Knife/stabbing 3. Blunt 4. Other 5. Unknown or Not assessed   2. No  3. Suspected but unclear  4. Unknown or Not assessed |  | H&P  Pediatrics  Social work  Psychology  Prior admissions in the medical record |
| **Prior suicide attempt, threats or self-harm** | This indicates if the patient has a history self-harm behavior, suicide attempts, threats or ideation. | 1. Yes (select all that apply)   1. Suicide attempt(s) 2. Self-harm behavior(s) (i.e. cutting) 3. Suicide threat(s) 4. Suicidal ideation   2. No  3. Suspected but unclear  4. Unknown or Not assessed |  | H&P  Prior ED notes  Psychiatry  Psychology  Social Work  Prior ED visits or admissions documented in the medical record |
| **Adverse experiences and/or exposures** | This indicates if the patient has experienced or is experiencing significant adverse or traumatic events. | 1. Yes (select all that apply)   1. Sexual assault or abuse 2. Physical violence 3. Exposure to or witness to violence (such as IPV or community violence) 4. Neglect 5. Psychological abuse 6. Loss of a parent 7. Parent incarceration 8. Parent or caregiver substance abuse 9. Homelessness or housing insecurity 10. Food insecurity 11. Major illness or injury 12. Parent separation or divorce 13. Being in child protective custody 14. Other major trauma   2. No  3. Suspected but unclear  4. Unknown or Not assessed | Please describe the experiences or exposures, if known. | H&P  Pediatrics  Psychiatry  Psychology  Social Work |
| **Firearm ownership and access** | This indicates if the patient has access to a firearm that they own, is in their home, or the home of a close friend or family member. | 1. Yes 2. Self (patient owns) 3. Family member or co-habitant in the home that the patient lives 4. Family member or individual close to the patient in another home 5. No 6. Suspected but unclear 7. Unknown or Not assessed |  | H&P  Pediatrics  Social Work  Psychiatry |

**Circumstances**

| **Variable** | **Definition** | **Options** | **Further Descriptions (Qualitative)** | **Common EMR Locations for Data** |
| --- | --- | --- | --- | --- |
| **Intent** | This indicates the intent of the shooting. | 1. Assault 2. Self-inflicted (suicide attempt) 3. Unintentional 4. Police-related shooting 5. Undetermined or Unknown 6. Not assessed |  | H&P  EMS documentation  ED note  Initial consult  Social work |
| **Setting of injury** | This indicates the type of setting that the injury took place in. | 1. Patient’s home 2. Other home or residence 3. Workplace 4. Car 5. Street or sidewalk 6. Woods or forest 7. Public space (i.e. mall, restaurant, bar) 8. Other 9. Unknown or unclear 10. Not assessed |  | EMS documentation  H&P  ED note  Social work  *There is an ICD-10 recorded for incident location, but these are not always accurate, specific and are sometimes missing when it can be identified in the record. |
| **Relationship to shooter** | This indicates the relationship of the shooter or perpetrator that is responsible for injuring the patient. | 1. Patient (self) 2. Someone known to patient (other than a significant other or family member) 3. Stranger 4. Significant other or ex-partner 5. Family member 6. Law enforcement 7. Other 8. Unknown or unclear 9. Not assessed   **Stranger* indicates the patient and/or witnesses saw the assailant and the suspect is not known to the patient  **Unknown or unclear* indicates the patient did not see the assailant or will not disclose the identity |  | H&P  ED note  Initial consult notes  Psychiatry  Social work |
| **Type of firearm used** | This indicates the type of firearm used to injure the patient. | 1. Handgun 2. Shotgun 3. Rifle 4. BB or pellet gun 5. Unknown or unclear 6. Not assessed |  | H&P  ED note  Initial consult note  Social work |
| **Owner of firearm used (suicide attempt or unintentional)** | This indicates who owned the firearm that was used to injure the patient. | 1. Patient (self) 2. Significant other 3. Other family member 4. Acquaintance, friend or colleague 5. Law enforcement 6. Unknown or unclear 7. Not assessed |  | H&P  ED note  Initial consult note  Pediatrics  Psychiatry  Social work |
| **How the firearm used was stored (suicide attempt or unintentional)** | This indicates how the firearm was was secured and stored at the time of injury. | 1. Locked in safe, lockbox or gun lock 2. Unlocked 3. Unknown or unclear 4. Not assessed | Describe the circumstances regarding where and how the firearm was accessed. For instance, if the firearm was kept loaded and accessed from a closet, side-table, automobile, etc. Or, if it was accessed while it had a safety device such as a safe, lockbox or gun lock. | H&P  ED note  Initial consult note  Pediatrics  Psychiatry  Social work |
| **Context of injury** | This indicates the context in which the injury was sustained. | **Assault: (select all that apply)**   1. Community violence 2. Bystander 3. Interpersonal altercation 4. Drug related 5. Intimate partner violence (direct or indirect victim) 6. Family violence 7. Mass shooting 8. Police shooting 9. Commission of a crime 10. Other 11. Unknown or unclear 12. Not assessed   **Self-inflicted: (select all that apply)**   1. Altercation 2. Intoxication 3. Declining mental illness 4. Cognitive impairment (other than acute intoxication) 5. Declining medical condition 6. Life stressors* 7. Murder-suicide 8. Other 9. Unknown or unclear 10. Not assessed   **Unintentional: (select all that apply)**   1. Handling or cleaning gun 2. Playing with gun 3. Hunting 4. Gun fell or discharged when presence of firearm was unknown 5. Celebration 6. Other 7. Unknown or unclear 8. Not assessed   **Life stressor* could include a divorce, loss of employment, economic distress, loss of a loved one, loss of a home or housing insecurity | Describe the circumstances documented for the events preceding and surrounding the time of injury. Such descriptions may include: patient was shot in a drive-by shooting related to retaliatory suspected gang activity, patient was shot by her husband after an argument and there is a history of IPV related violence and psychological abuse including prior injuries, and the patient shot himself after getting in an argument with his ex-girlfriend and was intoxicated but had no prior known ideation or mental illness. | H&P  ED note  Initial consult note  Pediatrics  Psychiatry  Social work |
| **Definitions for Assault Context:**  Community violence: Exposure to *intentional* acts of interpersonal violence committed in public areas by individuals who are not intimately related to the victim. Examples of this type of violence include gang disputes, drive-by shootings, bullying, and shootings that take place at school or in the neighborhood.  Bystander: The patient was unintentionally injured by a gunshot when another person was targeted for injury.  Interpersonal altercation: The patient got in an altercation, disagreement or dispute with a stranger, acquaintance or friend and was shot in the setting or as a result of a dispute.  Drug-related: The patient was shot in the setting of an illicit substance transaction or dispute.  Police shooting: The patient was shot by a law enforcement officer.  Commission of a crime: The patient was shot while reportedly committing a crime such as a robbery, assault, etc (not by a law enforcement officer).  Mass shooting: Shooting in which 4 or more people are shot.  Murder-suicide: The individual commits suicide or attempts to in the setting of immediately fatally injuring another individual  Family violence: The patient was shot by a member of the family that is not the intimate partner or ex-partner of the patient, nor was it related to IPV.  Intimate partner violence (IPV): The patient was shot by a current or former intimate partner (spouse, fiancé, girlfriend/boyfriend) or ex-partner, or the patient was shot in an IPV-related shooting (i.e. a child, friend or family member). | | | | |

**Early Functional Status and Healthcare Needs at the time of Discharge**

| **Variable** | **Definition** | **Options** | **Further Descriptions (Qualitative)** | **Common EMR Locations for Data** |
| --- | --- | --- | --- | --- |
| **Functional status** | This indicates the maximum level of functional status the patient achieves at the time of discharge. | 1. Basic activities of daily living 2. Instrumental activities of daily living 3. Advanced activities of daily living 4. Unknown or unclear   **Basic:** ability to perform basic self-care activities (eating, dressing, bathing, toileting)  **Instrumental:** ability to live independently (housework, cooking, laundry, managing transportation)  **Advanced:** ability to fulfill societal, community, and family roles and participate in recreational or occupational tasks |  | Physical and occupational therapy  Discharge summary |
| **Rehabilitation/post-discharge needs** | This indicates what rehabilitation services or post-hospital care the patient is recommended to have at the time of discharge. | Indicate all that apply:   1. None 2. Inpatient rehabilitation 3. Outpatient physical therapy 4. Outpatient occupational therapy 5. Outpatient speech therapy 6. Outpatient rehabilitation medicine 7. Other therapies 8. Unknown or unclear |  | Physical and occupational therapy  Case management / social work  Discharge summary |
| **Home health needs** | This indicates what home health needs are recommended at the time of discharge. | Indicate all that apply:   1. None 2. Home health nursing 3. Wound care 4. Infusion therapy 5. Other 6. Unknown or unclear |  | Physical and occupational therapy  Case management / social work  Discharge summary |
| **Psychosocial ancillary services** | This indicates what additional services are recommended at the time of discharge for psychosocial needs. | Indicate all that apply:   1. None 2. Social work or case manager 3. Child protective services 4. Psychologist 5. Psychiatry 6. Hospital and community based violence intervention programs 7. Intimate partner violence services 8. Other 9. Unknown or unclear |  | Social work  Psychology  Psychiatry  Discharge summary |
